# Measurable residual disease in Japanese patients with relapsed or refractory chronic lymphocytic leukemia treated with venetoclax

**DOI:** 10.1007/s12185-023-03646-3

**Published:** 2023-08-15

**Authors:** Koji Izutsu, Kazuhito Yamamoto, Koji Kato, Takayuki Ishikawa, Noriko Fukuhara, Yasuhito Terui, Ilseung Choi, Sumiko Okubo, Natsumi Ogawa, Mizu Sakai, Yasuko Nishimura, Brenda Chyla, Yan Sun, Dai Maruyama

**Affiliations:** 1 Department of Hematology, National Cancer Center Hospital, Tokyo, Japan; 2 Department of Hematology and Cell Therapy, Aichi Cancer Center, Aichi, Japan; 3 Department of Hematology, Oncology and Cardiovascular Medicine, Kyushu University Hospital, Fukuoka, Japan; 4 Department of Hematology, Kobe City Medical Center General Hospital, Hyogo, Japan; 5 Department of Hematology, Tohoku University Hospital, Miyagi, Japan; 6 Department of Hematology Oncology, Cancer Institute Hospital, Japanese Foundation for Cancer Research, Tokyo, Japan; 7 Department of Hematology, Saitama Medical University Hospital, Saitama, Japan; 8 Department of Hematology, National Hospital Organization, Kyushu Cancer Center, Fukuoka, Japan; 9 AbbVie GK, Osaka, Japan; 10 AbbVie GK, Tokyo, Japan; 11 AbbVie, Inc, North Chicago, IL USA

Dear Editor,

Monitoring of measurable residual disease (MRD) can assess depth of response and predict prognosis in patients with chronic lymphocytic leukemia (CLL) treated with immuno-chemotherapy or targeted therapies such as venetoclax monotherapy or venetoclax plus anti-CD20 monoclonal antibodies, which effectively eradicate CLL cells. In the phase 3 MURANO trial comparing venetoclax plus rituximab (VenR) against bendamustine plus rituximab (BR) in patients with relapsed or refractory CLL (rrCLL), patients in the VenR arm had better progression-free survival (PFS) and a higher rate of undetectable MRD (uMRD; less than 1 × 10^−4^) in peripheral blood (PB) at end of treatment than those receiving BR, regardless of presence of high-risk biomarkers [1]. Moreover, uMRD at the end of treatment in patients in the VenR arm was associated with longer PFS and overall survival compared with patients with high MRD + (≥ 1 × 10^−2^). Moreover, the median time from MRD conversion to clinical progression was 25.2 months, suggesting that early intervention before clinical progression in patients with MRD conversion might be a clinical option that merits testing in clinical trials. Fixed-duration therapy using venetoclax is being introduced for first-line treatment of CLL. Consequently, monitoring of MRD is increasingly important for CLL management, but reports on MRD monitoring in these patients remain rare in Japan. Here, we report MRD monitoring results of our phase 1/2 study of venetoclax with/without rituximab in patients with rrCLL/small lymphocytic lymphoma (SLL) (M13-834, NCT02265731) [2].

Study design, eligibility criteria, and dosing of M13-834 were as previously reported [2]. In brief, M13-834 was an open-label, multi-center phase 1/2 study of venetoclax monotherapy or combination therapy in Japanese patients with hematological malignancies. Six patients with rrCLL/SLL were enrolled in Arm B (phase 1 study of venetoclax monotherapy) and six with rrCLL only were enrolled in Arm D (phase 2 study of VenR). In both arms, venetoclax was administered with a weekly stepwise ramp-up in dose, followed by a dose of 400 mg/day. In Arm D, rituximab 375 mg/m^2^ was administered on day 1 of week 6, followed by 500 mg/m^2^ every 4 weeks until day 1 of week 29. Efficacy was evaluated according to the International Workshop on CLL consensus guidelines.

Samples for MRD measurements were prospectively obtained from PB at baseline (both arms), day 1 of week 24 (Arm B), day 1 of week 30 and every 12 weeks thereafter (Arm D), and within 3 months after complete remission (CR), after CR with incomplete blood count recovery, or when radiographic partial response (PR) criteria were first met (both arms). MRD was measured using the clonoSEQ^®^ assay, a next-generation sequencing-based assay that identifies rearranged IgH, IgK, and IgL receptor gene sequences. The limit of detection and the limit of quantitation of this assay were reported as 1.903 and 2.390 malignant cells [3]. Depending on the amount of genomic DNA input, this assay can detect MRD at least at the 10^–6^ level (< 1 in 1 million cells). MRD data were available for two patients (Patients 1 and 2) in Arm B and four patients (Patients 3–6) in Arm D. However, PB samples were unavailable for the other six patients due to SLL in two patients, discontinuation of the study because of adverse events and progressive disease in two patients, and unreported reasons in the remaining patients. We evaluated achievement of uMRD or MRD4 as defined by < 1 × 10^−4^ MRD (< 1 cell/10,000 leukocytes or < 0.01%) [4].

Tumor responses and MRD are presented in Table 1. All four patients receiving VenR achieved CR and uMRD. Meanwhile, two patients receiving venetoclax monotherapy did not achieve CR and uMRD, and their best response was PR. In Patients 3–6, the time to uMRD was 23 (range: 14–40) weeks. This study had several limitations. Because the number of patients with available MRD monitoring data was small, it remains unclear whether uMRD correlated with PFS or other endpoints in this population, or whether these results can be extrapolated to other MRD monitoring methods, such as allele-specific polymerase chain reaction and flow cytometry [4].

**Table 1:** Tumor response and measurable residual disease (MRD) in the peripheral blood of each patient

Patient number	Ven (Arm B)		VenR (Arm D)			
	#1	#2	#3	#4	#5	#6
Treatment duration (weeks)	41	76	118	115	60	124
Best overall response rate	PR	PR	CR	CR	CR	CR
Time to response (weeks)	6	6	6	14	6	6
Time to CR/CRi (weeks)	N/A	N/A	30	42	30	30
Duration of CR/CRi (weeks)	N/A	N/A	> 88	> 73	> 30	> 94
MRD level	MRD3	MRD3	MRD4	MRD4	MRD4	MRD4
Time to MRD4 (weeks)	N/A	N/A	40	26	14	20
Duration of MRD4 (weeks)	N/A	N/A	48	> 76	> 39	> 90
Lowest MRD level, log(MRD), within each period						
0 to 26 weeks	−3.1	−3.9	−3.8	−5.4	−5.6	−6.3
27 to 52 weeks	N/A	N/A	−4.2	−6.2	−6.0	−6.8^*^
53 to 78 weeks	N/A	N/A	−4.4	−6.8	−6.2	−6.9^*^
79 to 104 weeks	N/A	N/A	−4.4	−5.7	N/A	−6.8^*^
105 to 130 weeks	N/A	N/A	−3.9	N/A	N/A	−6.5^*^

In Arm B, MRD was measured at screening, 24 weeks after initial dosing, and within 3 months after radiographic PR, CR, or CRi. In Arm D, MRD was measured at screening, 30 weeks after initial dosing and then every 12 weeks during the study period, and within 2–3 months after radiographic PR, CR, or CRi. Additional measurements were performed as needed in both arms. Tumor responses were evaluated according to the International Workshop on Chronic Lymphocytic Leukemia consensus guidelines

CR, Complete remission; CRi, CR with incomplete blood count recovery; MRD, Measurable residual disease (in peripheral blood); MRD4, < 1 × 10^−4^ MRD; MRD3, < 1 × 10^−3^ MRD; N/A, Not applicable; PR, Partial response; Ven, Venetoclax monotherapy, VenR, Venetoclax plus rituximab combination therapy

* Below the lower limit of quantification

In conclusion, MRD monitoring using the clonoSEQ^®^ assay is feasible in Japanese patients with rrCLL who received venetoclax. uMRD was achieved in all patients treated with VenR with available MRD data.

## Data Availability

AbbVie is committed to responsible data sharing regarding the clinical trials we sponsor. This includes access to anonymized, individual and trial-level data (analysis data sets), as well as other information (e.g., protocols and Clinical Study Reports), as long as the trials are not part of an ongoing or planned regulatory submission. This includes requests for clinical trial data for unlicensed products and indications. This clinical trial data can be requested by any qualifed researchers who engage in rigorous, independent scientifc research, and will be provided following review and approval of a research proposal and Statistical Analysis Plan (SAP) and execution of a Data Sharing Agreement (DSA). Data requests can be submitted at any time and the data will be accessible for 12 months, with possible extensions considered. For more information on the process, or to submit a request, visit the following link: https://www.abbvieclinicaltrials.com/hcp/data-sharing/.
